# Pyrene-Imidazole Based Aggregation Modifier Leads to Enhancement in Efficiency and Environmental Stability for Ternary Organic Solar Cells

**DOI:** 10.3389/fchem.2018.00578

**Published:** 2018-11-28

**Authors:** Hui Lin, Xiaoyang Du, Lijuan Li, Caijun Zheng, Silu Tao

**Affiliations:** School of Optoelectronic Science and Engineering, University of Electronic Science and Technology of China, Chengdu, China

**Keywords:** pyrene-imidazole, π-π stacking, organic solar cells, fullerene aggregation, environmental stability

## Abstract

A novel pyrene-imidazole derivative (PyPI), which can form effcient π-π stacking in solid film, has been utilized in organic solar cells (OSCs). The stacking of small a molecule PyPI can facilitate a charge transfer and suppress fullerene aggregation. As a result, PTB7-Th: PyPI: PC_71_BM based ternary OSC exhibits a high power conversion efficiency (PCE) of 10.36%, which presents a 15.88% increase from the binary device (8.94%). Concurrently, the ternary OSC shows a much better thermal and light illumination stability. Under continuous 60°C annealing for 3 h, in atmosphere, the device still remains at 94.13% efficiency more than the pristine state, while the control device remains at 52.47% PCE. Constant illumination under Air Mass (AM) 1.5G irradiation (100 mW cm^−2^) in atmosphere, the PCE of OSC remains at 72.50%. The high conversion efficiency and excellent environmental stability of the PyPI based ternary OSC, has narrowed the gap between laboratory investigation and industrial production.

## Introduction

Solvent processed organic solar cells (OSCs) have attracted extensive attention for their superiority in achieving high power conversion efficiency (PCE), low fabrication cost and fascinating potential application in flexible electronics (Kaltenbrunner et al., 2012; Chen et al., 2014; Cui et al., 2017; Bergqvist et al., 2018; Cheng et al., 2018; Zhang H. et al., 2018). Generally, existing OSCs can be classified as binary, tandem, and ternary structures. In a traditional bulk heterojunction binary system, although the matched donor and acceptor can form a bicontinuous network interpenetrating structure to accelerate exciton dissociation and charge collection, the narrowed absorption bonds limited the further optimization on the device efficiency (Huang et al., 2017; Xu and Gao, 2018) (Li et al., 2018; Liu et al., 2018). To compensate for the shortcomings of the absoption spectrum, tandem OSCs composed of two or more subcells were fabricated to capture more photons and yielded more photon-generated carriers than conventional single-junction OSCs (Chen S. et al., 2017; Che et al., 2018; Zhang Y. et al., 2018). However, the complexity of the device structure and intricate interface,caused an increase in the device fabrication cost (Ameri et al., 2009; Kumari et al., 2017).

Compared with tandem OSCs, ternary strategy, which adds a third component to a binary system to broaden the absorption spectrum and promotes an interaction between the donor and acceptor, is an emerging and promising candidate for high performance OSCs with a simple device structure (Lu et al., 2015; Liu et al., 2016; Nian et al., 2016; Chen Y. et al., 2017; Xu et al., 2017). For ternary OSCs, the screening of the third component (either polymers or small molecules) is crucial. Challenges still remain in polymer materials as their purification and reproducibility are poor, further more, the chemical structure of polymers difficult hard to confirm. Other than polymer materials, small molecule materials have a simpler synthetic route and it is easy to obtain high purity. Additionally, the small molecules always have a mono-dispersed structure, with controlled energy levels and negligible batch to-batch variations (Chen et al., 2013; Roncali et al., 2014). Therefore, ternary OSCs using a small molecule as the third component have attracted increasing attention and have a great potential for achieving high-performance OSCs (Park et al., 2016; Chen Y. et al., 2017; Kumari et al., 2017; Zhang et al., 2017). As for the current ternary OSCs, the short-circuit current density's (J_SC_s) are still limited by the narrowed absorption strength, which is because of the thickness of active layers are confined to about 100 nm (Yang et al., 2015; Zhang J. et al., 2015; Zhang Y. et al., 2015; Zhang et al., 2017; Gasparini et al., 2016). That is to say, finding a way to enhance the J_SC_s of current OSCs is essential.

In this work, a novel small molecule PyPI (9,10-diphenyl-9H-pyreno[4, 5-d]imidazole) has been utilized to construct ternary organic solar cells. The small molecule PyPI can form efficient π-π stacking in a solid film, which is beneficial to accelerate a charge transfer in an active layer. Furthermore, the addition of PyPI can suppress fullerene aggregation and enhance device stability. For device fabrication, polymer PTB7-Th (poly(4,8-bis(5-(2-ethylhexyl)thiophen-2-yl)benzo[1,2-b;4,5-b0] dithiophene-2,6-diylalt-(4-(2-ethylhexyl)-3-fluorothieno[3,4-b]thiophene-)-2-carboxylate-2-6-diyl), and fullerene PC_71_BM ([6,6]-phenyl-C_71_-butyric acid methyl ester) was respectively used as a donor and acceptor. This polymer-fullerene system has great compatibility and is widely used in the field of organic solar cells. After precise modulation, 10% PyPI doped ternary OSC showed a high PCE of 10.36% with an optimized J_SC_ of 19.26 mA/cm^2^, which exhibited a 15.88% enhancement from the control device. In addition, after continuous thermal annealling at 60 or 80°C in atmosphere for 180 min, the PCE of the device can also be kept above 89.01%, while the control device remained at 36.96% PCE. The remarkable thermal stability is among the best of fullerene based OSCs. After continuous light illumination under 100 mW/cm^2^, the PyPI-containing ternary device also reveals optimized stability with a small efficiency roll-off of 27.23%, while the control device exhibits a huge PCE roll-off of 58.79%. The improvement in environmental stability demonstrates that the addition of the small molecule PyPI indeed supprsses the aggregation of fullerene.

## Experimental section

### General information

The materials and solvents utilized in the device fabrication and measurements were received from commercial suppliers without further purification. PTB7-Th (wt. 145,000) and PC_71_BM was purchased from 1-Material and American Dye Source. PEDOT:PSS was purchased from Xi'an p-OLED Technology Corp. MoO_3_ and LiF received from Luminescence Technology Corp. All solvents used in the device fabrication process originated from Sigma-Aldrich or Alfa Chemical.

### Device fabrication and measurement

Conventional inverted device structures were used for the binary and ternary OSCs. ITO covered glasses with a sheet resistance of 15 Ohm per square was utilized as the substrates for these devices. The substrates were ultrasonically cleaned, in the order of deionized water, ethyl alcohol, acetone and ethyl alcohol. Before transport layers were deposited, the substrates were dried by a nitrogen blow. For the transport layer, ZnO precursor solution was formed by dissolving 110 mg of zinc acetate (Zn(CH_3_COO)_2_•2H_2_O) and 31 mg of ethanolamine (NH_2_CH_2_CH_2_OH) in 1 ml of 2-methoxyethanol (CH_3_OCH_2_CH_2_OH) and stirring at room temperature over night. As for the active layers, Donor and acceptor were blended with the ratio of 1:1.5, where the donor contained PTB7-Th and the small molecule PyPI, with varying proportions and maintained at a total concentration of 10 mg/ml. Chlorobenzene (CB) was used as the solvent for each of the devices. 3.0 vol% 1,8-diiodooctane (DIO) was added in the mixture as an additive. The active layer precursor solution was stirred in a nitrogen filled glove box for 24 h. For OSC fabrication, ZnO nano-particles were formed by spin-coating the precursor solution with 5,000 rpm for 30 s, after which the substrates were transferred to a heating stage and annealed immediately at 200°C for 1 h in atmosphere. The substrates were the transferred to the glove box and the active layer precursor solutions were spin-coated onto the ZnO buffer layer to yield an uniform film (~120 nm). After that, the substrates were transferred to a vacuum deposition chamber and when the pressure of the chamber reached 5 × 10^−4^ Pa, 10 nm MoO_3_ and 150 nm, Ag were evaporated at a rate of 0.5 and 3 Å/s, subsequently. An active area of 2.3 mm^2^ was formed by a shadow mask.

### Experimental measurements

UV absorption spectra and photoluminescence spectra of monomeric and blend films were recorded by a Hitachi U-3010 UV-VS spectrophotometer and a Perkin-Elmer LS50B Luminescence spectrophotometer, respectively. The HOMO/LUMO energy level was determined by cyclic voltammetry with a CHI600E electrochemical analyzer. Nitrogen saturated DCM was used as a solvent with 0.1 mol/L tetrabutylammonium hexa?uorophosphate as the supporting electrolyte.

The performance of the solar cells were measured by AM 1.5G simulated sunlight (Newport Oriel Sol3A Simulator, 100 mW/cm^2^) with a Keithley 2,400 source meter instrument. EQE properties were determined by a QEX10 Quantum Efficiency Measurement System (PV Measurements, Inc.). The thicknesses were calibrated by a AMBIOS-XP2 step profilometer. The surface morphologies of the binary and ternary blend films were determained by an atomic force microscope (AFM) under ambient conditions. All the films were formed on ZnO coved substrates. The molecule aggregation and formed domain size were observed by a transmission electron microscopy (TEM) scanning-probe SPM system (Hitachi TEM system) under 100 kV in “Ceshigo Research Service, www.ceshigo.com.”

## Results and discussions

### Characterization and optical properties

**Figure 1:**
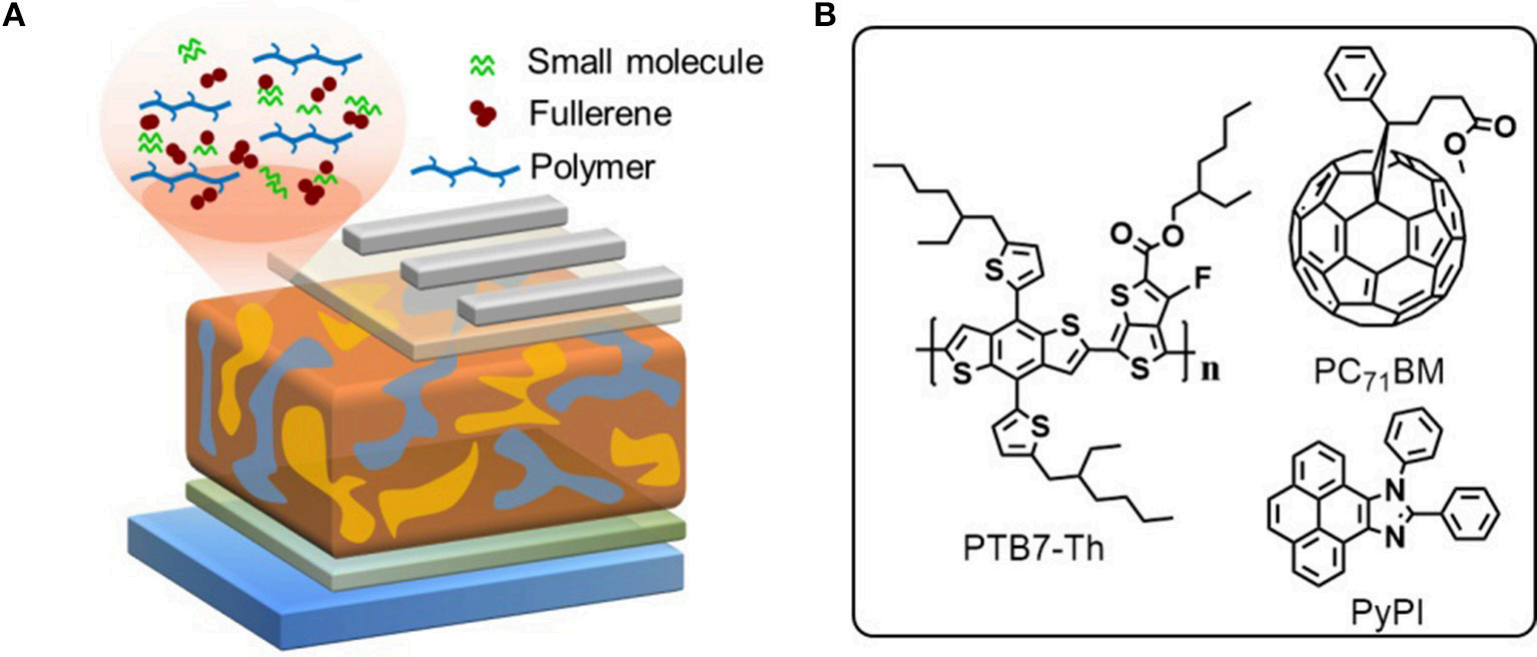
**(A)** device structure and schematic diagram of the ternary OSC; **(B)** materials structure of the used materials.

**Figure 2:**
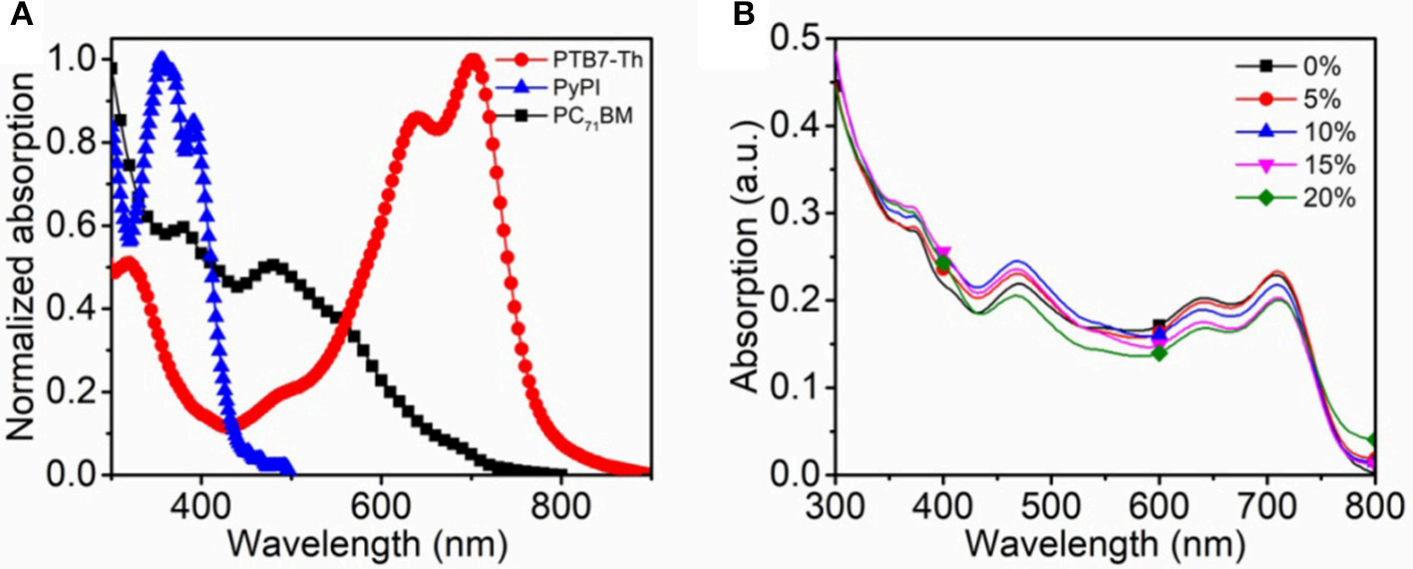
**(A)** normalized absorption spectra of PTB7-Th, PC_71_BM, and PyPI in neat film; **(B)** absorption spectra of the control and ternary blend films with different components of PyPI.

The chemical structures of used materials in the device as well as device structure are shown in Figure 1. It is well-known that the fullerene aggregates in the interspace between the stacked clearances of polymers, while the small molecule PyPI can form efficient π-π stacking in the clearance in a polymer, which can suppress this inferior phenomenon as indicated in the diagram of Figure 1A (Liu et al., 2017). We tested through cyclic voltammetry, that the LOMO and HOMO levels of the small molecule PyPI was −2.37 and −5.42. Figure 2A reveals the absorption spectra of PTB7-Th, PC_71_BM and the small molecule PyPI, the maximum absorption peak for PTB7-Th centered at 704 nm and that of PyPI film was located at 355 and 391 nm. As in the blend films, the increase of small molecule contents along with the absorption intensity, gradually increased in the range of short wavelength, while the intensity declined in turn around long wavelengths of 600–800 nm as shown in Figure 2B. This is because the contents of PTB7-Th have been decreased along with the increase of PyPI. Furthermore, PyPI contained films shows a small red-shift in long wavelength absorption, suggesting an enhanced stacking order in polymers, which may be caused by the positive interaction between the small molecule and PTB7-Th.

### Photovoltaic performance

To evaluate the contribution of the π-π stacking effect on the device performance, the small molecule PyPI was utilized as the third component to fabricated ternary OSCs. Device structure was performed as follows: ITO/ ZnO (20 nm) /active layer (120 nm) /MoO_3_ (10 nm)/Ag (150 nm) and is shown in Figure 1A, a ZnO transport layer was formed with 20 nm thickness and the active layer was prepared in a glove box with 120 nm thickness. The total donor concentration was kept at 10 mg/ml in CB solvent, where the doping ratio of PyPI was tuned from 0 to 20% in donors. The weight ratio of donor: acceptor was maintained at 1:1.5. DIO was used as a solution additive with a volume ratio of 3%. To estimate the average OSC performance, about 20 samples were fabricated for each parameter.

**Figure 3:**
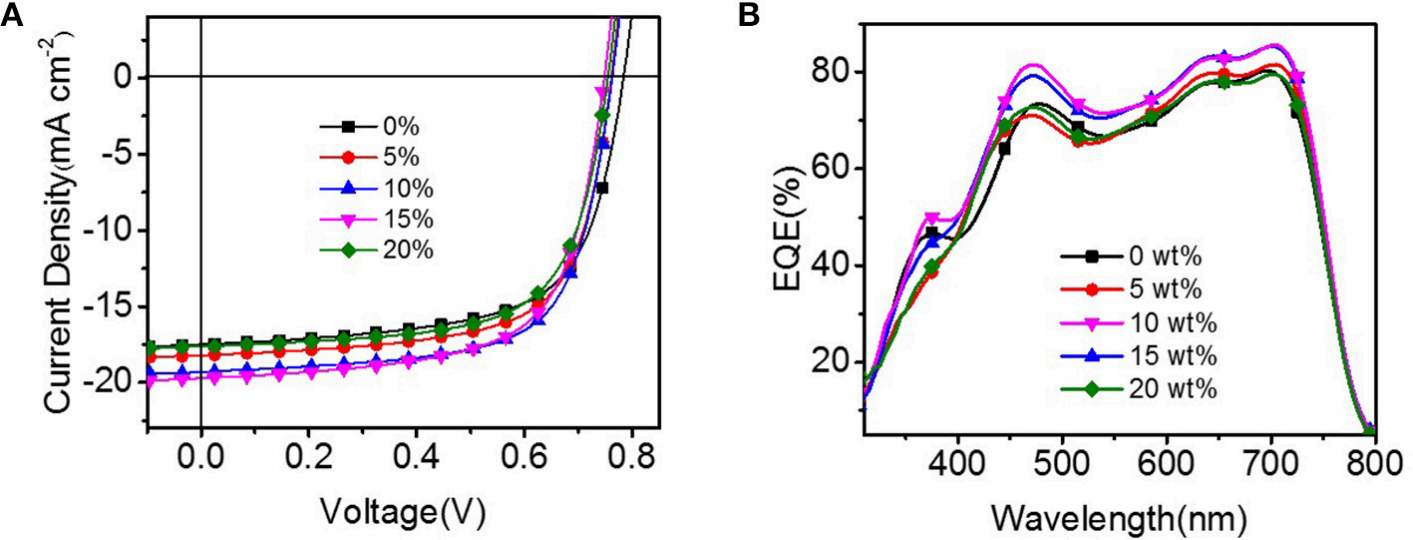
**(A)** J-V curves of the binary and ternary OSCs under AM 1.5G irradiation at 100 mW cm^−2^; **(B)** The EQE curves the binary and ternary OSCs.

The current density-voltage (J-V) characteristics of the binary and ternary OSCs are shown in Figure 3A, the corresponding device parameters are listed in Table 1. After rigorously optimizing the fabrication conditions, the binary OSC obtained a maximum PCE of 9.11%, along with a short-circuit current density (J_SC_) of 17.46 mA/cm^2^, an open-circuit voltage (V_OC_) of 0.78 V and a fill factor (FF) of 65.38%. Upon adding 5% to 20% PyPI into the binary systems, the device performances were observably improved. The highest PCE of 10.36% was achieved for PTB7-Th: 10% PyPI: PC_71_BM, which exhibited a maximum enhancement of 15.88% more than the control device. Concurrently, the J_SC_ value was enhanced from 17.46 to 19.26 mA/cm^2^, suggesting that the charge transfer was greatly improved when PyPI was doped in the binary system. The enhancement in current density is attributed to the stacking effect of PyPI that accelerates the charge transfer in the active layer. The FF improvement of ternary OSCs can be attributed to the optimizing film morphology as discussed below. When the concentration of the small molecule increased to 20%, the PCE decreased along with the degradation on J_SC_ and FF, which was mainly dominated by the overlarge domain size of the ternary OSC. EQE properties of the binary and ternary OSCs were measured to calibrate the high J_SC_s, and more detailed data are listed in Table 1. Figure 3B shows the EQE curves with a different PyPI component, and all devices exhibit a prominent photo-generated current response in the whole visible absorption region from 300 to 800 nm. Along with the doping ratio of PyPI increasing from 0 to 10%, EQEs present a remarkable enhancement both in short wavelength (300–400 nm) and long wavelength (500–800 nm). The improvement in a 300–400 nm absorption bond is attributed to the contribution of PyPI, while the enhancement in the long wave range of polymer (500–800 nm) may be attributed to the optimization of polymer crystallinity. Further added the doping ratio of PyPI to 20%, the device EQE showed huge degeneration, and device performance also decreased, as the excess added PyPI reduced the content of the polymer and decreased the donor/acceptor connections. As a result, the ternary OSC shows a reduced J_SC_ and FF.

**Table 1:** Performance summaries of PTN based ternary OSCs.

**PTB7-Th:PyPI:PC_71_BM**	**V_oc_ (V)**	**J_sc_ (mA/cm^2^)**	**J_calc_^*a*^ (mA/cm^2^)**	**FF(%)**	**PCE(average)^*b*^ (%)**
100:0:150	0.78	17.46	17.22	65.38	9.11 (8.94)
95:5:150	0.77	18.17	17.96	67.50	9.69 (9.34)
90:10:150	0.77	19.26	18.94	67.90	10.36 (9.97)
85:15:150	0.76	19.06	18.62	67.22	10.02 (9.72)
80:20:150	0.76	17.59	17.13	66.81	9.07 (8.87)

a *J_calc_ is calculated from EQE spectra*.

b *Statistical data obtained from 20 devices*.

### Recombination dynamics

**Figure 4:**
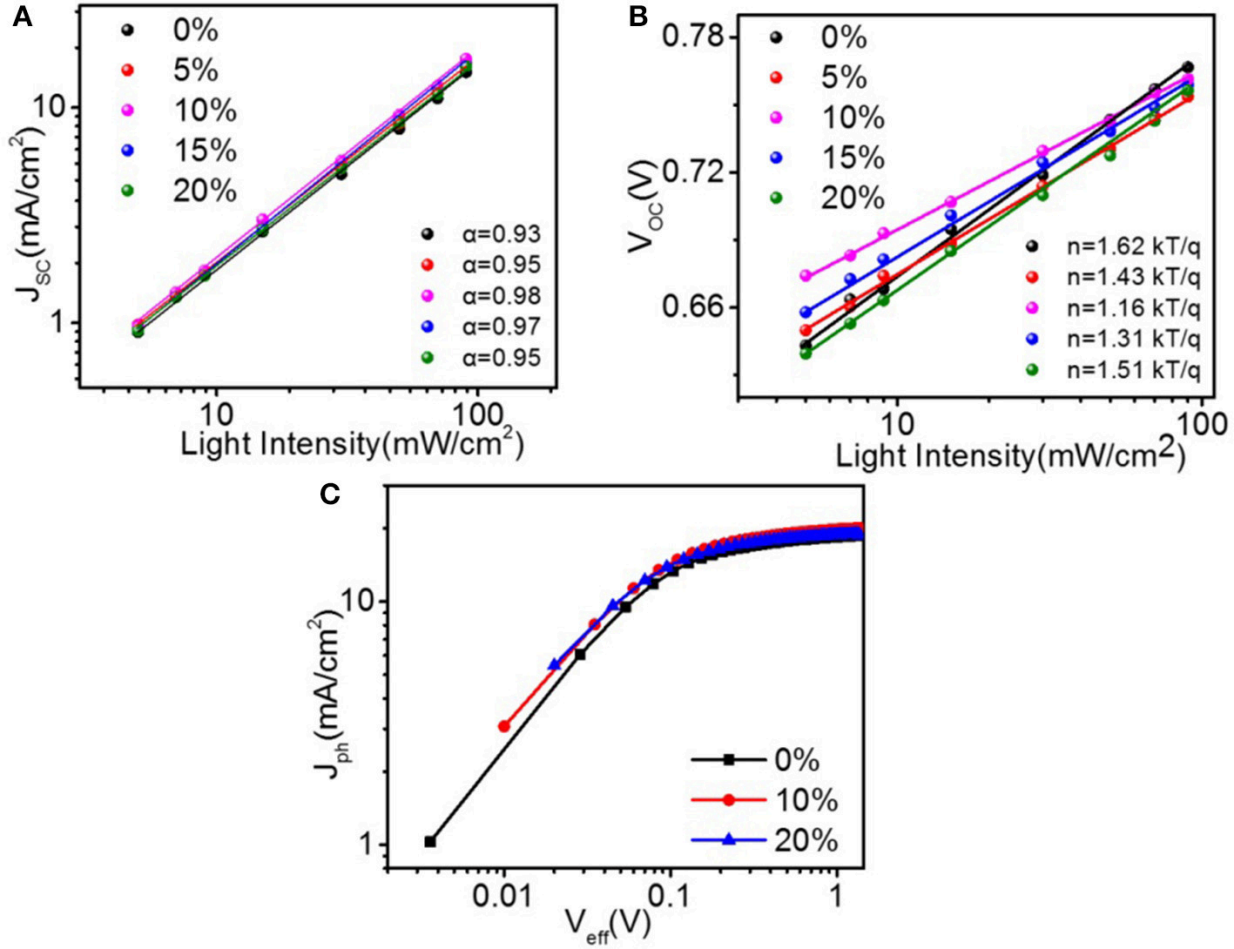
Light-intensity dependence of **(A)** J_SC_ and V_OC_ **(B)** for ternary OSCs with different amounts of PyPI. **(C)**J_ph_-V_eff_ curves of binary and ternary devices.

The charge generation and recombination dynamics' behavior in these solar cells were studied. *J*_*SC*_ and *V*_*OC*_ vs. light intensity (*P*_*L*_) plotted on logarithm coordinate with the linear fittings are shown in Figures 4A,B, respectively. In *J*_*SC*_-*P*_*L*_ measurements, the variation in *J*_*SC*_ as a function of *P*_*L*_ can be concluded as $J_{SC}\propto P_{0}^{\alpha }$, where α = 1 is indicative of the inexistence of bimolecular recombination in the film under short-circuit conditions. The α values of the solar cells with 0, 5, 10, 15, and 20 % PyPI were 0.93, 0.95, 0.98, 0.97, and 0.95, respectively, indicating very weak bimolecular recombination in these devices. Additionally, as shown in Figure 4B, the Voc of optimal device based on PTB7-Th:10 % PyPI: PC_71_BM shows a logarithmic dependence on *P*_*L*_ with a slope of 1.16 kT/q compared to that of 1.62 kT/q in the binary system. The slope value for the cells containing 5, 15, and 20 % PyPI were 1.43, 1.31, and 1.51 kT/q, respectively. The results suggested that the trap-assisted recombination was effectively alleviated by adding 10% PyPI. The characteristics of the photocurrent density (*J*_*ph*_) vs. the effective applied voltage (*V*_*eff*_) were then measured to further understand the charge generation and dissociation process in the ternary OSCs with different contents of PyPI. In theory, *J*_*ph*_ is defined as *J*_*ph*_ = *J*_*L*_ − *J*_*D*_, where *J*_*L*_ and *J*_*D*_ represent photocurrent density and dark current density, respectively. *V*_*eff*_ is defined as *V*_*eff*_ = *V*_0_ − *V*_*bias*_, where *V*_0_ is the voltage at which *J*_*L*_ is equal to *J*_*D*_ and *V*_*bias*_ is the applied bias voltage. As shown in Figure 4C, the exciton dissociation probabilities (*P*_*diss*_), which are determined by *J*_*SC*_/*J*_*sat*_, were calculated as 92.2, 95.4, and 93.1% for the PTB7-Th:PC_71_BM binary device and ternary devices with 10 % and 20% PyPI under the short-circuit condition, respectively. The higher *P*_*diss*_ demonstrates that the corresponding ternary device has more efficient exciton dissociation and charge extraction. These results indicated that the ternary blend system can restrain charge recombination and facilitate exciton dissociation when compared to the binary system, which corresponds to the device performance.

### Film morphology and molecule distribution

**Figure 5:**
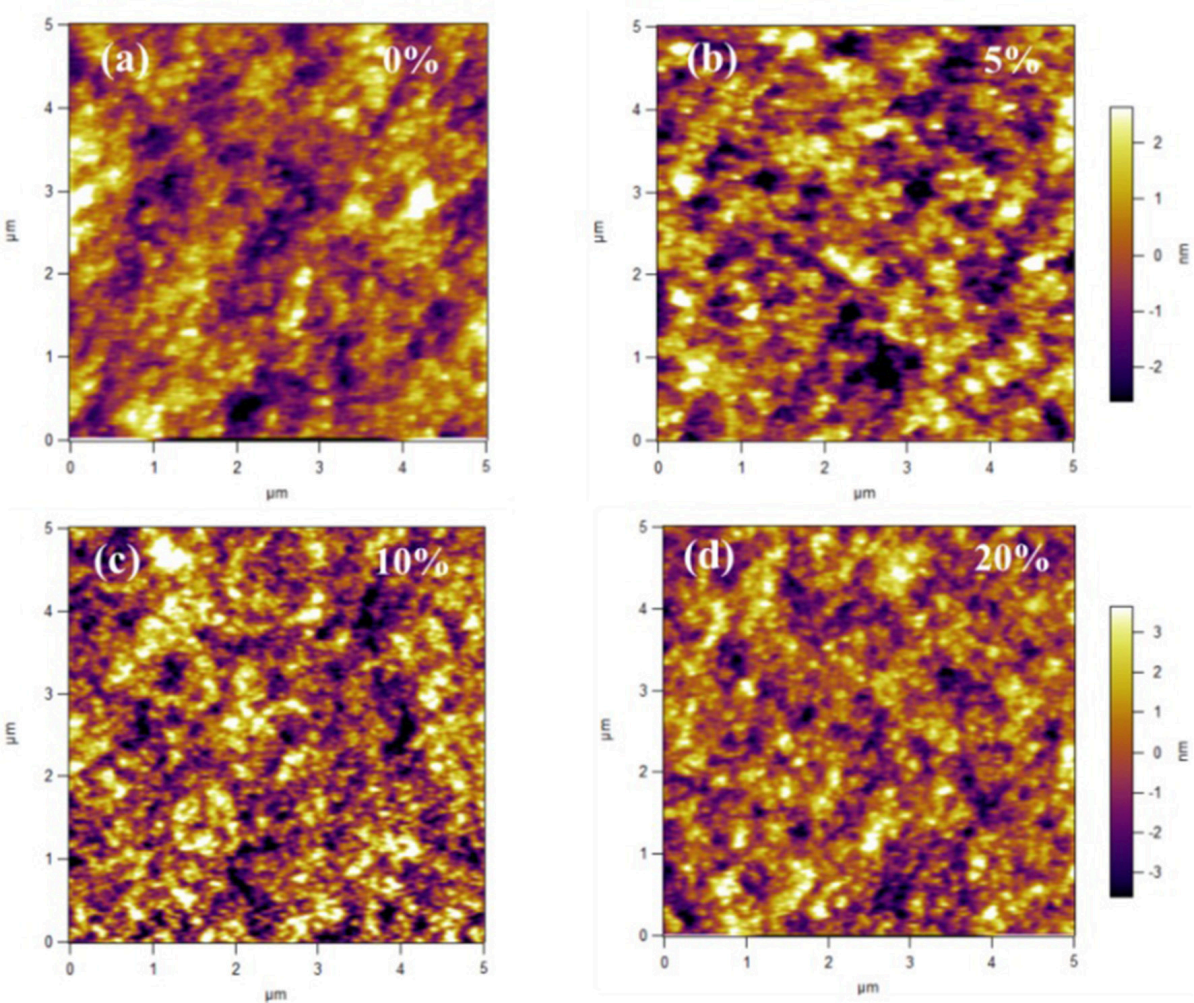
AFM images for **(a)** PTB7-Th:PC_71_BM; **(b)** PTB7-Th: 5%PyPI: PC_71_BM; **(c)** PTB7-Th:10%PyPI: PC_71_BM; **(d)** PTB7-Th:20%PyPI: PC_71_BM based film.

To explore the reasons why the small molecule PyPI can improve the performance of conventional machines, the surface topography and potential of the active layers of the ternary devices were examined by an atomic force microscope (AFM). As shown in Figure 5, after addition of 5 and 10% PyPI, the ternary blend films remained homogeneous and smooth with similar root-mean-square (RMS) values of 1.53 and 1.29 nm, respectively, which is much better than that of the control film (1.78). Obviously, the RMS value decreases as the PyPI content increases, which means that a suitable amount of PyPI can be well-incorporated into the PTB7-Th:PC_71_BM based control device and the surface morphology of the membrane can be optimized. When the PyPI content exceeded 10 wt% and increased to 20 wt%, the RMS increased to 1.81 due to the lower solubility of the small molecules at such a high doping ratio, resulting in a larger domain size. The formation of large areas reduced the contact interface between the donor and acceptor, which is the main reason for the lower J_SC_ and PCE of these ternary OSCs.

**Figure 6:**
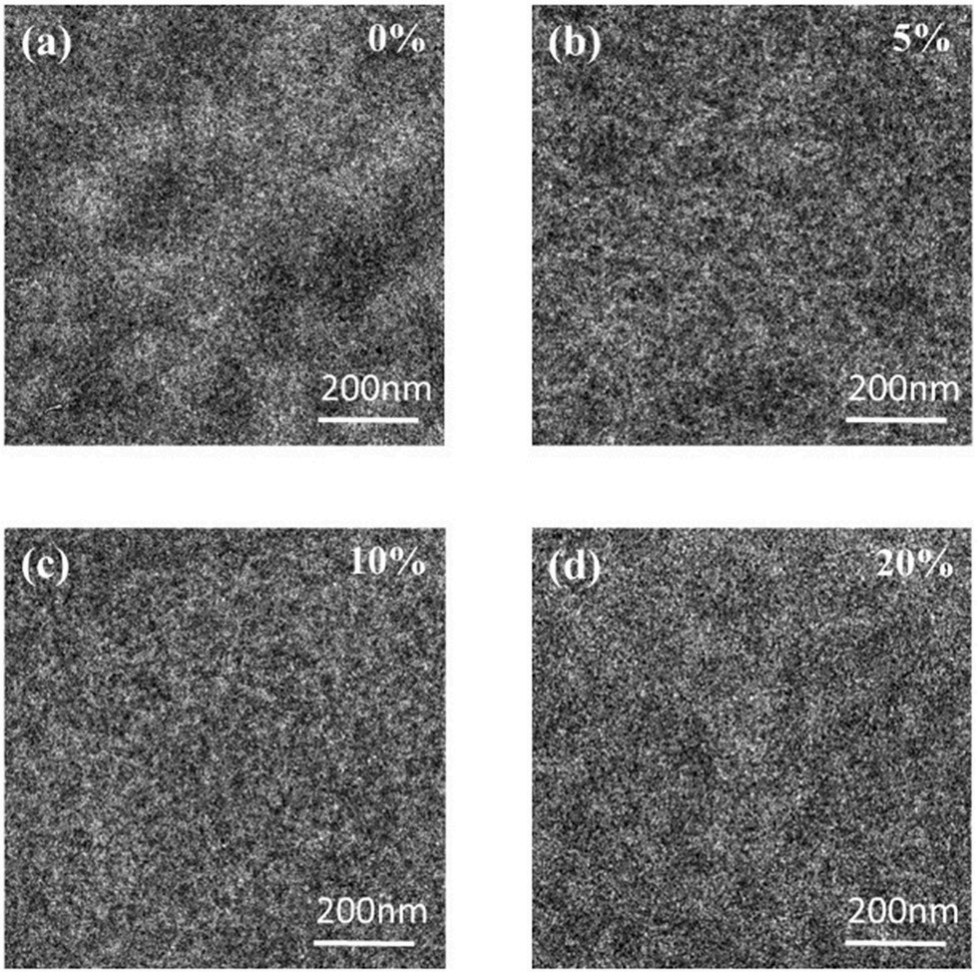
TEM images for **(a)** PTB7-Th:PC_71_BM; **(b)** PTB7-Th: 5%PyPI: PC_71_BM; **(c)** PTB7-Th:10%PyPI: PC_71_BM; **(d)** PTB7-Th:20%PyPI: PC_71_BM based film.

Since the AFM image only reflects the surface information of the film layer, the phase distribution is studied by TEM, and the images are shown in Figure 6. It is well-known that bright and dark areas correspond to rich PTB7-Th and PC_71_BM regions, depending on the electron density. After the addition of 5% PyPI (Figure 6b) to prepare a ternary blend film, a better phase separation was observed compared to the binary film. The ternary device with a 10% PyPI content showed a fairly uniform membrane morphology with a rich donor/acceptor interface (Figure 6c), resulting in higher J_SC_ and FF. The nanofiber network of PTB7-Th can be observed in the PTB7-Th:PC_71_BM blend membrane. In addition, a large number of randomly distributed large regions are obtained in the active layer. For ternary blend membranes, the nanoscale network becomes more pronounced as the PyPI content increases. In the ternary blend membrane, PyPI can modulate the PTB7-Th molecular alignment and optimize phase separation and enhance photon collection of the active layer.

### Environmental stability

**Figure 7:**
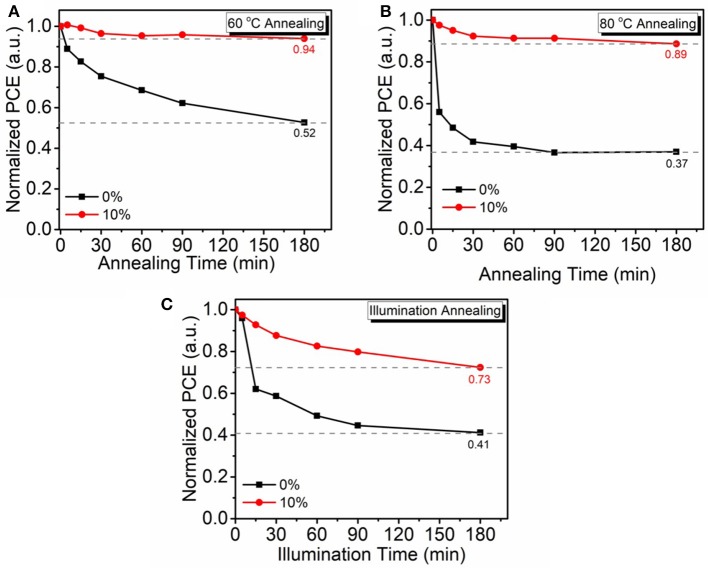
Non-encapsulated device stability annealed at 60°C **(A)**, 80°C **(B)**, and under AM 1.5G irradiation (100 mW cm^−2^), **(C)** in atmosphere.

It is well-known that the aggregation of fullerene is the dominant reason of OSC performance degeneration in atmosphere. As small molecule PyPI can form a positive π-π stacking to suppress fullerene aggregation and we speculate that the intruding PyPI in the fullerene based OSC can improve the devices environmental stability. To verify this deduction, device stability of the non-encapsulated OSCs was investigated through thermal and light irradiation annealing in atmosphere. Thermal annealing temperatures were set at 60 to 80°C, which is a practical operation temperature range for OSCs under AM 1.5G irradiation (100 mW/cm^2^). Figure 7A shows the thermal stability curves of control OSCs and 10% PyPI contained ternary OSCs that continuously annealed at 60 and 80°C in atmosphere for 180 min. After the annealing process, the PCE of ternary OSC remains at 94.13% of it pristine state, which is much higher than the control device (52.47%). Even more surprising, by further annealing the devices at 80°C for 180 min, PyPI based ternary OSCs also revealed a mild decay, as shown in Figure 7B, PyPI contained OSC still remained at 89.01% PCE, whereas the control binary device only processed 36.96%. The superior thermal stability of the ternary OSC is mainly ascribed to the addition of the small molecule which suppressed fullerene aggregation. Figure 7C shows the light illumination stability curves that were measured by continuously illuminating the PyPI based ternary device under 100 mW cm^−2^ AM 1.5G irradiation in atmosphere. The PyPI-containing ternary device revealed optimized stability with a small efficiency roll-off of 27.23%, while the control device exhibited a large PCE roll-off of 58.79%. The improvement in light illumination stability is mainly attributed to the introduction of the small molecule PyPI, restraining the aggregation of fullerene. The control device exhibited more severe efficiency degradation than PyPI based ternary OSC, which is attributed to the fact that fullerene can form dimers when exposed in light illumination (Fortunato et al., 2013; Wang et al., 2014; Heumueller et al., 2016). The dimeric fullerenes could significantly suppress charge transfer and deteriorate film morphology, resulting in declined device performance.

## Conclusion

A novel ternary organic solar cell system containing PTB7-Th: PyPI as the donor and PC_71_BM as the acceptor has been fabricated to enhance device efficiency and environmental stability. The small molecule PyPI can form efficient π-π stacking in a solid film, which is beneficial to accelerate charge transfer and suppress fullerene aggregation in the active layer. After rigorous modulation, 10% PyPI contained ternary OSC exhibited a high PCE of 10.36%, which presented a 15.88% enhancement from the control device. Furthermore, the ternary OSC showed excellent thermal and light illumination stability. Under thermal annealing for 3 h in atmosphere, the device remain at 94.13% efficiency, over pristine state, while the control device only remained at 52.47% PCE. In the condition of constant illumination under AM 1.5G irradiation (100 mW cm^−2^) in atmosphere, the PCE of OSC can remain at 72.50% PCE. The excellent performance of PyPI based OSC will stimulate the development of solar cells in practical production.

## Author contributions

All authors listed have made a substantial, direct and intellectual contribution to the work, and approved it for publication.

### Conflict of interest statement

The authors declare that the research was conducted in the absence of any commercial or financial relationships that could be construed as a potential conflict of interest.
